# Implementation of the Enhanced Recovery After Surgery (ERAS®) program in neurosurgery

**DOI:** 10.1007/s00701-023-05789-y

**Published:** 2023-09-09

**Authors:** Amani Belouaer, Giulia Cossu, Georgios E. Papadakis, John G. Gaudet, Maria-Helena Perez, Vivianne Chanez, Yann Boegli, Caroline Mury, David Peters, Valérie Addor, Marc Levivier, Roy Thomas Daniel, Nicolas Demartines, Mahmoud Messerer

**Affiliations:** 1 Department of Clinical Neuroscience, Service of Neurosurgery, Lausanne University Hospital (CHUV) and University of Lausanne, Lausanne, Switzerland; 2 Service of Endocrinology, Diabetology, and Metabolism, Lausanne University Hospital (CHUV) and University of Lausanne, Lausanne, Switzerland; 3 Department of Anesthesiology, Neurospinal Unit, Lausanne University Hospital (CHUV) and University of Lausanne, Lausanne, Switzerland; 4 Pediatric Intensive and Intermediate Care Units, Department of Pediatrics, Women-Mother-Child Department, Lausanne University Hospital (CHUV) and University of Lausanne, Lausanne, Switzerland; 5 Department of Anesthesiology, Pediatric Unit, Lausanne University Hospital (CHUV) and University of Lausanne, Lausanne, Switzerland; 6 Department of Visceral Surgery, Lausanne University Hospital (CHUV) and University of Lausanne, Lausanne, Switzerland

**Keywords:** Enhanced Recovery after Surgery (ERAS®), Pituitary adenoma, Pituitary neuroendocrine tumors, Transsphenoidal, Craniosynostosis, Neurosurgery, Pediatric

## Abstract

**Background:**

Over the past decade, Enhanced Recovery After Surgery (ERAS®) guidelines have been proven to simplify postoperative care and improve recovery in several surgical disciplines. The authors set out to create and launch an ERAS® program for cranial neurosurgery that meets official ERAS® Society standards. The authors summarize the successive steps taken to achieve this goal in two specific neurosurgical conditions and describe the challenges they faced.

**Methods:**

Pituitary neuroendocrine tumors (Pit-NET) resected by a transsphenoidal approach and craniosynostosis (Cs) repair were selected as appropriate targets for the implementation of ERAS® program in the Department of Neurosurgery. A multidisciplinary team with experience in managing these pathologies was created. A specialized ERAS® nurse coordinator was hired. An ERAS® certification process was performed involving 4 seminars separated by 3 active phases under the supervision of an ERAS® coach.

**Results:**

The ERAS® Pit-NET team included 8 active members. The ERAS® Cs team included 12 active members. Through the ERAS® certification process, areas for improvement were identified, local protocols were written, and the ERAS® program was implemented. Patient-centered strategies were developed to increase compliance with the ERAS® protocols. A prospective database was designed for ongoing program evaluation. Certification was achieved in 18 months. Direct costs and time requirements are reported.

**Conclusion:**

Successful ERAS® certification requires a committed multidisciplinary team, an ERAS® coach, and a dedicated nurse coordinator.

## Introduction

The Enhanced Recovery After Surgery (ERAS®) program aims to improve postoperative recovery by reducing the stress response to surgery [24] [25]. Back in 2001, a multidisciplinary group led by two surgeons, Ken Fearon and Olle Ljungqvist, launched the first ERAS® study group with the goal of improving outcomes after colon surgery, following the promising experience of Kehlet et al. [3]. After the publication of very encouraging initial results [12, 15, 21], they created the ERAS® Society in 2010 and started applying similar patient-centered, evidence-based, multimodal, and multidisciplinary ERAS® protocols to various other surgical procedures [1, 4, 5, 7, 9, 10, 36]. This led to significant improvements in patient care and recovery including reduced length of hospital stay, perioperative complications, and healthcare costs [13, 31, 36].

Despite the recent publication of ERAS®recommendations for patients undergoing lumbar spinal fusion [10], there are currently no certified ERAS® guidelines for cranial neurosurgery. Several neurosurgical teams have claimed successful implementation of “ERAS®-like” protocols for cranial surgery [33, 35]. However, these efforts to standardize cranial perioperative neurosurgical care have all been monocentric and have not reported compliance metrics, significantly limiting their applicability, reproducibility, and efficiency at all levels of care. As a result, they have not been certified by the ERAS® Society.

The authors set out to create and launch an ERAS® program for cranial neurosurgery that meets the ERAS® Society criteria [18, 23, 32]. The present paper summarizes the successive steps taken to achieve this goal within the Department of Neurosurgery at the University Hospital of Lausanne (CHUV), describes the challenges faced and how they were solved. This project started with two specific neurosurgical conditions, namely pituitary neuroendocrine tumors (Pit-NET) resected by a transsphenoidal approach and craniosynostosis (Cs) repair.

### The rationale of the implementation of ERAS® in the Department of Neurosurgery: historical background

The CHUV is the ERAS® birthplace in Switzerland. In 2011, the Visceral Surgery Department started the first Swiss ERAS® program for colon surgery. Five years later, the application of ERAS® principles has postoperative complications and shortened the length of hospital stay by an average of two to three days following colon surgery [26]. This has contributed to significant improvement in safety outcomes, patient satisfaction, and further reduction of healthcare costs [13, 19].

In 2020, a first meeting with ERAS® leaders at CHUV was organized to discuss the launch of an ERAS® neurosurgical program. The neurosurgical team quickly realized that in the absence of any preexisting ERAS® program for cranial surgery, they would have to set up new structured and detailed local ERAS® protocols that were likely to have a significant impact on daily patient management at all levels of care. They opted to gradually ease them in to let healthcare teams adjust and offer feedback. Rather than changing all perioperative protocols at once, they selected two neurosurgical procedures as targets for new patient-centered, multimodal, and multidisciplinary pathways.

## Steps to implement ERAS.® program (Fig. 1)

**Fig. 1:**
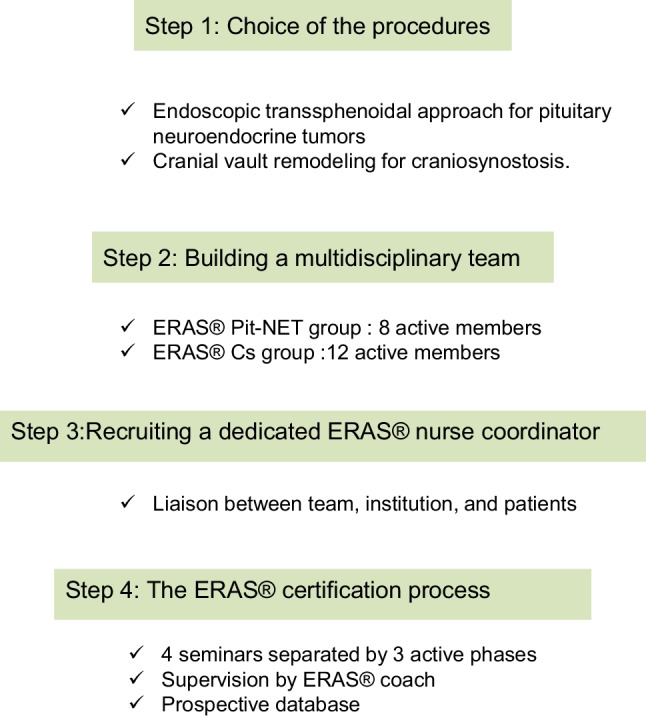
Steps to implement ERAS® Program

To ensure consistent delivery of outstanding healthcare, ERAS® protocols and guidelines place the patient at the center of his/her recovery, involve a multidisciplinary team coordinated by an ERAS® nurse coordinator, and are continuously monitored through an integrated audit system.

### Choice of procedures

To increase the chances of practical and successful application of local ERAS® protocols in their department, the neurosurgical team chose two appropriate procedures that met the following three key criteria:

i. Commonly performed in the institution
ii. Involved multiple healthcare teams before, during, and after surgery
iii. Reflected perioperative neurosurgical management in adult and pediatric settings in the institution

The two procedures that best met these criteria were the endoscopic transsphenoidal approach for pituitary neuroendocrine tumors (Pit NET) and cranial vault remodeling for craniosynostosis (CS).

#### Pituitary neuroendocrine tumors

Pituitary neuroendocrine tumors, also known as pituitary adenomas, are typically resected endoscopically through a mini-invasive, transsphenoidal approach. Up until the ERAS® process began, existing management protocols were inconsistently applied, causing perioperative care to vary significantly between cases. The authors identified that protocol deviations originated from multiple sources. Clearly, no single action would be able to dramatically improve compliance. Instead, a complete overhaul involving all teams was required.

#### Craniosynostosis

Craniosynostosis is a congenital pathology in which the bones in a newborn’s skull fuse too early before full growth of the brain. Consequently, this creates skull deformity and adversely affects neurocognitive development. Collaboration and communication between multiple teams is essential for the surgical management of this pathology. Without a consistent communication between pediatricians, intensive care team, neurosurgeons, and parents, important information can be lost. This can result in delayed refeeding, mobilization, or inappropriate management.

### Building a multidisciplinary team

After selection of the two neurosurgical procedures to test the local ERAS® protocols, the neurosurgical team built two groups that included key personnel at all levels of care for both Pit-NET transsphenoidal surgery and Cs repair. They selected members based on their motivation to participate and their experience with each chosen procedure.

ERAS® Pit-NET group included 8 active members: 3 neurosurgeons, 1 endocrinologist, 1 anesthesiologist, 2 clinical nurses, and one ERAS® nurse coordinator.

ERAS® Cs group included 12 active members: 3 neurosurgeons, 2 pediatric intensive care physicians, 2 anesthesiologists, 4 clinical nurses, and one ERAS® nurse coordinator.

In each group, every member contributed to the design and implementation of new evidence-based and practical ERAS® clinical protocols.

### Recruiting a dedicated ERAS® nurse coordinator

The ERAS® nurse coordinator plays a central role in the multidisciplinary team [2]. He/she is responsible for planning meetings, keeping ERAS® groups on track, and actively promoting ERAS® principles among caregivers. His/her main goal is to maximize compliance in daily clinical practice [2]. In short, the nurse coordinator must act as a liaison within the ERAS® groups and inside the department and institution. Experience with medical research is helpful, as he/she is often called to enter, edit, and retrieve data from local and/or remote databases. The ERAS® nurse coordinator also gives feedback that is used to update protocols. This position is critical for successful implementation and ongoing evaluation of the ERAS® protocol. The Department of Neurosurgery provided funds to hire a part-time nurse coordinator and fully supported her, both logistically and administratively.

### The ERAS® certification process

**Fig. 2:**
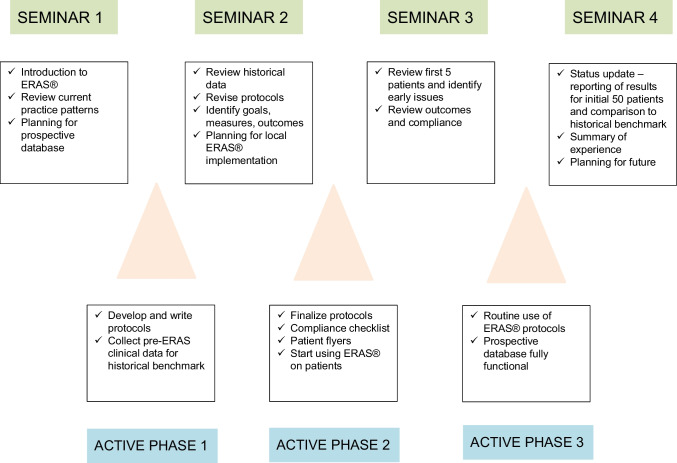
The ERAS® certification process

Certification by the ERAS® Society requires several criteria: compliance ≥ 70% after implementation, decrease of length of stay, and complications. Moreover, all members of the multidisciplinary team have successfully completed a structured certification process that is centered on a series of 4 seminars spread over a period of 8 to 10 months. Between each seminar, there is an active phase where the discussion topics of each seminar are implemented by local ERAS® groups (Fig. 2). An ERAS® coach coordinates and monitors the entire process that culminates with the ERAS® certification. The multidisciplinary team that successfully completes this process is identified as ERAS® Qualified Units. Hereafter are details on each step of the process.

#### First seminar: reviewing current practice and selecting variables of interest

During the first seminar, members of the multidisciplinary team collectively review current perioperative management procedures. This step must be completed before any revision is contemplated. After assessment of current practice patterns, the multidisciplinary teams can start working on developing new “ERAS®-minded” protocols. The expertise of each member is key to ensuring that emerging ERAS® protocols describe the role of each caregiver and are applicable at all levels of care within the institution. Equally important is the development of a prospective database that can be used to evaluate the new protocols. Accurately evaluating new protocols in comparison to historical results requires identifying the most useful data and ERAS® metrics to measure and record prospectively.

#### Active phase 1: revising current practice and collecting historical data

After the first seminar, each member took some time to critically reappraise how healthcare is delivered for both selected surgical procedures, focusing specifically on how to optimize perioperative management in their field of expertise and based on recent recommendations in the literature. The key steps identified for improvement in the first active phase included the following:

In the Pit-NET group,

- Preoperative fasting was shortened and the endocrinologist updated perioperative hormonal replacement protocols.
- Perioperative radiological exams and ophthalmological check-up were scheduled at specific timepoints.
- The neurosurgical team streamlined surgical prepping and draping and standardized the surgical technique, using uninostril approaches [6, 28] (except for large or giant tumors) [8] and performing a reconstruction for CSF leakage with autologous abdominal fat, with no use of lumbar drains or naso-septal flaps. No nasal packing was used to allow early breathing through the nose.
- The anesthesiologist revised all anesthetic protocols, focusing specifically on pain management and fluid therapy, that was limited to 1500 ml during the first 4 h of surgery. Invasive measures were limited to selected cases: namely a urinary catheter was used only in surgeries expected to last more than 3 h, while an arterial line was limited to ACTH—or GH-secreting PitNET.
- Recommendations on postoperative non-invasive ventilation, antithrombotic and antimicrobial prophylaxis were standardized: mechanical and medical antithrombotic measures were started early during surgery and the first day after surgery, respectively, while the antibiotic therapy was limited to 24 h [11, 16, 22, 34].
- Early mobilization and feeding represented two important key points in the post-operative management to obtain an early discharge that was fixed 3 days after surgery (except in case of complication).

In the Cs group,

- The preoperative fasting was minimized according to the age of the child and ranged between 2 and 6 h.
- The neurosurgical team updated the perioperative radiological diagnostic strategy according to the current recommendation, adding systematic preoperative computed tomography with fine bone sections to identify subclinical craniosynostosis and other features that may affect the surgical technique [27, 30].
- The pediatric team started offering genetic counseling to all patients, not just those with syndromic or multiple craniosynostosis.
- The pediatric anesthesiology and intensive care teams worked together to standardize perioperative sedation and analgesia protocols, as well as blood product and fluid management. A particular effort was made to maintain a temperature between 36.5 and 37.5° C and to limit blood loss during surgery through the systematic administration of tranexamic acid.
- The surgical technique was also standardized, along with the assessment of the aesthetic outcomes [29].
- Systematic pain assessment through the use of the FLACC scale was an important point added in the early postoperative management, along with a controlled administration of opioids. Again, an early mobilization and feeding were introduced as important items to enable a fast recovery of our patients.

During the first active phase, the local ERAS® groups also retrospectively identified 30 consecutive patients who underwent Pit-NET and 30 consecutive patients with Cs repair surgery prior to implementation of any ERAS® protocols. They extracted clinical data to establish a historical benchmark.

#### Second seminar: lessons from other ERAS® groups and from the historical cohort

The second seminar was a time for reflection and exchange for the multidisciplinary team. The group discussed with ERAS coach and other ERAS teams the results based on the analysis of historical data. Opportunities for improvement had been identified during the first active phase and discussed in the seminar. Lessons learned from historical data, from other ERAS® programs, and guidance by the ERAS® coach helped to build and solidify the important steps of new “ERAS®-minded” protocols.

#### Active phase 2: drafting and testing the initial protocol

During the second active phase, the local ERAS® groups used all the knowledge and experience acquired up until this point to draft initial versions of the Pit-NET and Cs protocols. There are several examples available on the ERAS® website that provided useful guidance [4, 7, 22]. Whenever necessary, they also reached out to the ERAS® coach for advice. As recommended by the ERAS® Society, they met on a weekly basis during the drafting phase to discuss the protocol and reach a consensus. Debated points were settled according to a targeted and thorough analysis of the literature until a recommendation was approved by all team members.

In each group, all members had an opportunity to revise any part of the protocol. At the end, all members approved the protocols for both neurosurgical procedures.

At this stage, they had also to establish the main goals of the novel protocols and determine their compliance parameters. The following supporting documents were created to inform patients and help measure compliance.

- *Patient booklet* was given to all patients (or their parents) scheduled to undergo Pit-NET or Cs repair surgery. These documents included information on the goals of the ERAS® program and how it was going to impact the level of care they receive. The patient booklet solidified guidance and instructions provided by the ERAS® nurse coordinator. Importantly, it explains the active role of the patient/parents of the patient during the perioperative period. Finally, it contains contact information to ask questions and facilitate feedback. Once initial versions of the protocols and supporting materials were validated by the hospital “Patient Information Commission,” the authors started prospectively enrolling patients.
- *The patient logbook:* this document is built around a calendar, where patients can record how they feel both physically and mentally. It serves as reminders of daily tasks and goals, thereby keeping patients engaged in their own care and letting them monitor their progress during the immediate postoperative phase.
- *Compliance checklist:* the local ERAS® group produced documents listing all preoperative, intraoperative, and postoperative compliance metrics for each selected neurosurgical procedure. Continuous compliance monitoring is essential to determine whether even the most evidence-based ERAS® protocol is—and remains—applicable and beneficial to clinical practice.

#### Third seminar: identifying early issues and starting the dynamic ERAS® process

During the third seminar, first 5 patients enrolled in each group were reviewed. The session was designed to identify early performance and compliance issues in the presence of the ERAS® coach. The local ERAS® groups discussed outcomes such as complications, lengths of hospital stay, or causes for re-admission. Importantly, they also reviewed compliance statistics to pinpoint specific organizational or communication problems. ERAS® certification is a dynamic process, whereby perioperative management must be periodically reviewed and revised in order to improve and remain up to date.

#### Active phase 3: fine-tuning the protocol

During the third and final active phase, the local ERAS® groups continued actively enrolling patients in the Pit-NET and Cs ERAS® programs. They kept meeting weekly or biweekly to motivate each other and discuss problems encountered before, during, or after surgery at all levels of care. Their primary focus was on how to increase and maintain compliance with the protocols. By the end of this phase, the protocols were becoming standard of care in their institution.

#### Forth seminar: continuing the dynamic ERAS® process and meeting targets

The fourth and last seminar aimed to review the performance and compliance metrics once the first 50 patients had been enrolled for each pathology. In each group, they discussed the clinical data and compliance rates to the protocol. At this point, they compared outcomes in both the prospective and the historical cohorts to fully measure how much progress they had made.

To be certified, ERAS® programs must achieve and maintain compliance rates of at least 70%. ERAS® programs must also work transparently, providing open access to protocols, support materials, as well as performance and compliance metrics. ERAS® Qualified Units must continue to apply, monitor, and periodically revise protocols. To remain certified, ERAS® nurse coordinators or data manager must routinely enter all data into the ERAS® audit system.

## Specific roles

### The ERAS® nurse coordinator

As described above, the ERAS® nurse coordinator plays a central role as a liaison officer between all levels of care within the institution. Here are listed the specific responsibilities for patients/parents enrolled in an ERAS® program.

First and foremost, the ERAS® coordinator acts as a patient guide and counselor. He/she encourages them to actively engage in their own care by seeking information and giving feedback. The ERAS® nurse coordinator initially meets patients preoperatively to go over various aspects of the ERAS® program, but also to address more general or sensible issues that may be easier to discuss with a nurse than a physician, such as the return to normal activities, assistance for self-care, or logistical/scheduling conundrums. In doing so, the ERAS® nurse coordinator establishes a relationship based on trust, the cornerstone of compliance.

At the end of the consultation, a tour of the department is performed so that the patient can be familiarized with the hospital premises where they will stay during the hospitalization. The ERAS® nurse coordinator remains available throughout the hospital stay, and 1 week after discharge, he/she calls patients to collect follow-up outcome data.

### The ERAS® patient

The ERAS® philosophy is patient-centered and ERAS® programs are designed to encourage patients to play an active role at all stages. Improving patient education of their pathology and treatment increases compliance and satisfaction [17, 20].

#### Preoperative implication

During the preoperative phase, patients receive information in multiple formats. Instructions are provided in person by the ERAS® nurse coordinator during the preoperative consultation. Patients have access to a wealth of information compiled in a booklet specifically designed to clarify why they are having surgery and what to expect before, during, and after surgery. The booklet also contains useful recommendations on how to get ready for surgery, how to communicate with caregivers, and where to find more details if necessary.

#### Post-operative implication

During the postoperative phase, patients are encouraged to communicate with the healthcare team, as supported by literature data [14]. The patient logbook is designed to facilitate this task. Once discharged home, patients are encouraged to use the”CHUV@home” smartphone application to track their progress and recovery. This application is built around structured questionnaires that patients are invited to fill out twice a day (once a day for children) for a total of 10 days. Patients can also use the institutional mobile application “CHUV@home” to submit questions directly to the healthcare team. Trained nurses collect answers and respond to patients, following specific protocols written by specialists. If any red flags are identified, the patient is quickly referred to the appropriate medical team. This allows early diagnosis of post-operative complications and keeps patients in charge of their own care.

### Challenges and costs associated with the ERAS® certification process

#### Indirect costs: time and human resources

As illustrated, the ERAS® certification process is time-consuming. It brings together personnel from multiple professional environments. Making room in their busy schedules on a weekly basis is not an easy task. Without a doubt, this is the main challenge that the authors faced over the months that led to their program receiving certification.

It took 18 months in our department to meet the criteria of an ERAS® Qualified Unit. During that time, 6 physicians and 2 nurses spent a total of 793 h and 122 h, respectively, working on the ERAS® Pit-NET project. At the same time, 7 physicians and 4 nurses spent a total of 1037 h and 244 h, respectively, working on the ERAS® Cs project. The ERAS® nurse coordinator spent a total of 1342 h coordinating the two projects during those 18 months.

#### Direct costs: seminars, access to the database, and dedicated nurse

As part of an institution that is recognized as a Center of Excellence by the ERAS® Society, the implementation was made in “internal.” Similarly, the authors did not have to pay for access to the ERAS® database since there was no cranial neurosurgical ERAS® database. In other circumstances when an ERAS® database does exist, the service provider (Encare) charges maintenance and security fees. However, the authors had to pay the RedCap fees to create a secured data base. The amount was 304 CHF.

The hiring of an ERAS® nurse coordinator was the main direct source of expense. The Department of Neurosurgery agreed to finance a part-time (50%) contract fully dedicated to the ERAS® project. Over the course of 18 months, this amounted to a total of 81,903.78 CHF. Minor additional expenses included equipment purchases (computer, telephone) as well as travel to ERAS® meetings. This has to be regarded as an investment in favor of improved outcome.

## Next step: going global

After focusing on adult transsphenoidal pituitary neuroendocrine tumor resections and pediatric craniosystosis repair, the authors plan to apply ERAS® principles to more neurosurgical procedures, aiming progressively to include all cranial surgical procedures in the department.

The ultimate goal is to establish international ERAS® guidelines for the perioperative management of neurosurgical patients within the ERAS Society.

## Conclusion

ERAS® principles have changed the authors’ vision on perioperative management. In addition to keeping patients engaged in their own care, they have reshaped the perioperative mindset by focusing not only on performance, but also on applicability at all levels of care. In this narrative review, the authors presented how they completed the ERAS® certification process in the Department of Neurosurgery, a discipline where no ERAS® guidelines where available. They also described what adjustments they had to make, which challenges they faced at each step, and the costs required in order to successfully implement a novel ERAS® neurosurgical program.

## Data Availability

Not applicable.
